# Expression of an IRF-3 fusion protein and mouse estrogen receptor, inhibits hepatitis C viral replication in RIG-I-deficient Huh 7.5 cells

**DOI:** 10.1186/1743-422X-8-445

**Published:** 2011-09-21

**Authors:** Luyu Yao, Xiaobo Yan, Huijia Dong, David R Nelson, Chen Liu, Xiaoyu Li

**Affiliations:** 1 Division of Gastroenterology and Hepatology, Department of Medicine, University of Florida-Jacksonville, FL 32206, USA; 2 Department of Pathology, University of Florida-Gainesville, FL 32610, USA; 3 Department of Medicine, University of Florida-Gainesville, FL 32610, USA; 4 Department of Neurology, Second University Affiliated Hospital, Harbin Medical University, Harbin, 150081, China

## Abstract

Interferon Regulatory Factor-3 (IRF-3) plays a central role in the induction of interferon (IFN) production and succeeding interferon-stimulated genes (ISG) expression en route for restraining hepatitis C virus (HCV) infection. Here, we established a stable Huh7.5-IRF3ER cell line expressing a fusion protein of IRF-3 and mouse estrogen receptor (ER) to examine IFN production and anti-HCV effects of IRF-3 in retinoic acid inducible-gene-I (RIG-I) deficient Huh 7.5 cells. Homodimerization of the IRF-3ER fusion protein was detected by Western blotting after treatment with the estrogen receptor agonist 4-hydrotamoxifen (4-HT) in Huh7.5-IRF3ER cells. Expression of IFN-α, IFN-β, and their inhibitory effects on HCV replication were demonstrated by real-time polymerase chain reaction (PCR). Peak expression of IFN-α and IFN-β was achieved 24-hours post 4-HT treatment, coinciding with the appearance of phosphorylated signal transducer and activator of transcription (STAT) proteins. Additionally, HCV viral replication declined in time-dependent fashion. In previous studies, a novel IFN-mediated pathway regulating expression of 1-8U and heterogeneous nuclear ribonucleoprotein M (hnRNP M) inhibited HCV internal ribosomal entry site (IRES)-dependent translation. When expression of ISGs such as 1-8U and hnRNP M were measured in 4-HT-treated Huh7.5-IRF3ER cells, both genes were positively regulated by activation of the IRF-3ER fusion protein. In conclusion, the anti-HCV effects of IRF-3ER homodimerization inhibited HCV RNA replication as well as HCV IRES-dependent translation in Huh7.5-IRF3ER cells. The results of this study indicate that IRF-3ER homodimerization is a key step to restore IFN expression in Huh7.5-IRF3ER cells and in achieving its anti-HCV effects.

## Introduction

Hepatitis C virus infection causes chronic liver diseases, cirrhosis, and hepatic cellular carcinoma (HCC) with 170 million people worldwide and 4 million people in the United States reportedly infected (CDC, 1998). In addition to its global health problem [1], future projections suggest that HCV related mortality will increase 2-3-fold over the next decade [2] with more than 180 billion US dollars estimated total social economic cost in the United States [3]. The standard treatment of chronic HCV is anti-viral therapy with IFN and ribavirin (RBV) but no HCV vaccine available. Despite additional chemotherapeutics is on hand for treatment of genotype I HCV patients recently, the anti-viral mechanisms of IFN-based therapies are not well defined, but most likely involve the activation of host innate immunity to limit HCV replication.

During microbial infection, the recognition of microbial components is mediated by host-specific cellular pathogen-recognition receptors (PPRs). PPRs are members of the toll-like receptor (TLRs) family and are localized either to cellular plasma (TLR4 for lipopolysaccharide (LPS) and viral envelops) or endosomal membranes (TLR3 for dsRNA, TLR7/8 for ssRNA and TLR9 for DNA [4-7]). Conversely, intracellular dsRNA is also recognized by the RIG-I cytosolic RNA helicase or melanoma differentiation associated gene (MDA)-5 [8]. RIG-I RNA helicase was found to be an essential mediator of anti-HCV effects due to its binding to un-capped 5'-end and 3'-end HCV dsRNA, triggering host innate immunity [9].

IFNs bind to the IFN-α/β receptor (IFNAR) in either an autocrine and/or paracrine manner to initiate a positive feedback loop that results in the production of more type I IFNs. IFNARs trigger the activation of the JAK/STAT pathway to phosphorylate the STAT proteins. The STAT transcription factors associated with IRF-9 to form a heterotrimeric complex, IFN-stimulated gene factor 3 (ISGF3), initiating the transcription of IFN-stimulated genes (ISGs) and inhibiting the different stages of virus replication and eliciting an anti-viral state in the host [10,11]. During HCV infection, these anti-viral effects include the inhibitory effects on host and HCV translation [12,13], regulation of cellular proliferation and apoptosis [14], regulation of adaptive immunity [15,16], and recruitment of NK cells to the site of infection [17,18] to clear HCV infection by inhibiting HCV gene expression and HCV replication. Patients with cleared HCV infection without IFN-based treatment provides strong evidence for the host innate immune response during acute HCV infection [19,20].

In order to study the direct anti-HCV response of IRF-3 activation, an inducible Huh7.5-IRF3ER cell line was established in RIG-I deficient Huh 7.5 cells that allow IRF-3 protein homodimer formation in a cytokine/receptor-independent fashion. Huh 7.5 cells are a highly adapted and poorly differentiated hepatoma cell line that lacks the ability to produce detectable interferon-α/β when infected with HCV JFH-1 virus [21]. Therefore, Huh7.5-IRF3ER cells is an adequate system to study the downstream molecular events of IRF-3 activation and establishment of a subsequent anti-HCV state without RIG-I activation in Huh 7.5 cells.

## Materials and Methods

### Plasmids

A mammalian expression vector, pTIRF3ER, was constructed as a fusion protein of the IRF3 gene (51.6 Kd) [22] and C-terminal sequences of the mouse estrogen receptor (310 a.a.) [23] in the pEF6/V5-His TOPO^® ^TA vector (Invitrogen, Carlsbad, CA). The plasmid pJFH-1 contains a full-length HCV genomic cDNA [24]. The plasmid pRL-HL is a dicistronic construct that mediates Cap-dependent and HCV IRES-dependent translation [25]. Synthetic 4-hydroxytamoxifen (4-HT) was purchased from Sigma (Saint Louis, MO) and dissolved in ethanol as a 5 mM stock solution.

### Cell lines

Human hepatoma Huh 7.5 cells [26] were grown in Dulbecco's modified Eagle's medium (Invitrogen). To establish the Huh7.5-IRF3ER cell line, Huh 7.5 cells were transfected with the plasmid pTIRF3ER and Lipofectin (Invitrogen). Blasticidin (Invitrogen) (10 μg/ml) was used for the clone selection 24-hours after transfection. Medium was changed every 3 days with fresh Blasticidin until day 14, at which time, positive clones were propagated. The clones were amplified and IRF-3ER dimer formation was measured by Western blotting after 4-HT treatment.

### Detection of IRF-3ER dimers, p-STAT1 (S727), p-STAT3 (Y705), and 1-8U protein by Western blotting

Huh7.5-IRF3ER cell monolayers were washed in phosphate buffered saline (PBS) post 4-HT treatment with protease inhibitor cocktail (Sigma). Preparation of Huh7.5-IRF3ER cell lysates was performed as reported previously [27]. Cellular lysates were separated by sodium dodecylsulfate polyacrylamide gel electrophoresis (SDS-PAGE) (6% gel for IRF-3ER dimers; 8% gel for p-STAT1 (S727) and p-STAT3 (Y705)). Western blotting was carried out as previously reported [27] with antibodies for actin (Santz Cruz Biotechnology, Inc., Santa Cruz, CA), p-STAT3 (Y705) (Cell Signaling, Boston, MA), p-STAT3 (Y705) (Cell Signaling), and STAT1 (Santz Cruz). Western blotting of STAT1 and STAT3 proteins were performed with the same PVDF membrane used for detection of p-STAT1 (S727) and p-STAT3 (Y705) after stripping the blot (Bio-rad stripping buffer).

### HCV JFH-1 stocks and HCV infection

Preparation and titration of HCV JFH-1 virus was reported previously [24]. For examining anti-HCV effects, Huh7.5-IRF3ER cells were incubated with 0.5 MOI JFH-1 HCV for 14 days to achieve fully infected Huh7.5-IRF3ER monolayer cells [28]. The Huh7.5-IRF3ER cells were then treated with 4-HT for 72, 48 and 24 hours prior to collecting total cellular RNA. Huh7.5-IRF3ER cells without 4-HT treatment for 72 hours were used as control. Total RNA was isolated for detecting HCV RNA by real-time PCR.

### Detection of IFN-α and IFN-β in Huh7.5-IRF3ER cells

Huh7.5-IRF3ER cells were treated with 4-HT for 72, 48, and 24 hours prior to collecting cellular lysates. Control is Huh7.5-IRF3ER cells that did not receive 4-HT treatment for 72 hours. Total cellular RNA was isolated for detecting IFN-α or IFN-β RNA by real-time PCR.

### Real-Time PCR assay

Total cellular RNA was isolated from infected Huh7.5-IRF3ER monolayers by Trizol (Invitrogen). First-strand cDNA were synthesized from 1 μg total cellular RNA by reverse transcription (20 μl of reaction volume). Superscript II (200 U reverse transcriptase per reaction) and a RT-PCR kit (Invitrogen) was used to prime with oligo (dT) 12-18 for first-strand synthesis according to the manufacturer's instructions. Taqman primers were obtained from Applied BioSystems. Reactions were conducted in a 96-well MyiQ cycler (Bio-Rad, Hercules, CA). Fluorescence was monitored during every PCR cycle at the annealing step. The primers for HCV JFH-1 are: forward, 5'-CGGAATTGCCGGGAAGAC-3'; reverse, 5'-CAAATGGCCGGGCATAG AG-3'; FAM probe, 5'-CTTTCTTGGATAAACCC-3'. The primers for IFN-α are: forward, 5'- GGGATGAGGACCTCCTAGACAAATT-3'; reverse, 5'- ACACAGGCTTCCAAGTCA TTC-AG-3'; FAM probe, 5'- CTGCACCGAACTCTAC-3'. The primers for IFN-β are: forward, 5'-TGGCTGGAATGAGACTATTGTTGAG-3'; reverse, 5'-CAGGACTGTCTTCA GATGG-TTTATCT-3'; FAM probe, 5'-CCTCCTGGCTAATGTC-3'. GADPH primers were purchased from the Applied Biosystems. PCR was performed with the following conditions: 50°C, 2 min; 95°C, 10 min; (95°C, 15s; 60°C, 1 min) for 40 cycles. Relative RNA level indicates statistical quantification of altered RNA levels from these cellular lysates with different primers. Samples were run in triplicate and the results were analyzed using the Bio-Rad iQ5 software; means ± the standard error of the mean are shown.

### Luciferase assays

Huh7.5-IRF3ER cells were cultured in 6-well plates and transfected with the plasmid pRL-HL and lipofectamine 2000 (Invitrogen). After 24-hours of transfection, Huh7.5-IRF3ER cells were treated with 4-HT for 96, 72, and 48 hours prior to preparing cell lysates. Control Huh7.5-IRF3ER cells were incubated for 96 hours in the absence of 4-HT. All samples were analyzed for luciferase activity using the Dual-Luciferase Reporter Assay System Kit (Promega, Madison, WI) in triplicate. The translation efficiency was calculated as a proportion of control (100%).

### Statistical analysis

Different cellular lysates were collected for analysis of luciferase activity or relative RNA level from Huh7.5-IRF3ER cells with special treatment. Results of these studies are expressed as means ± standard deviation (SD).

## Results

### Dimerization of IRF-3ER fusion protein induced by 4-HT in Huh7.5-IRF3ER cells

**Figure 1:**
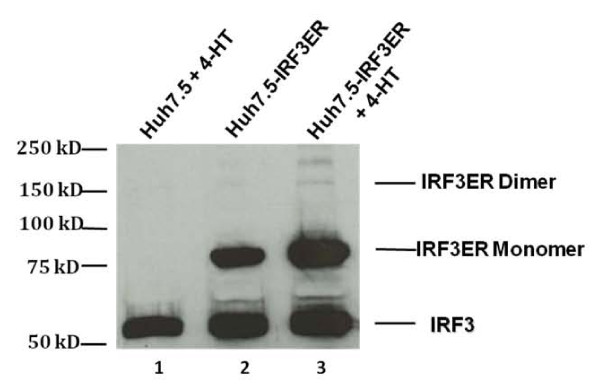
**Dimerization of the IRF-3ER fusion protein in Huh7.5-IRF3ER cell line**. Huh7.5-IRF3ER and Huh 7.5 cells were treated with or without 4-HT. Total cellular protein was extracted and analyzed for Western blotting with anti-IRF3 antibody. Lane 1, Huh7.5 cells treated with 4-HT. Lane 2, Huh7.5-IRF3ER cells without 4-HT treatment. Lane 3, Huh7.5-IRF3ER cells treated with 4-HT.

Activation of IRF-3 or IRF-7 is a critical step during virus infection, promoting the most potent type I IFN production. Previous studies showed the constitutively active forms (serines replaced by phosphomimetic aspartate amino acids) of human IRF-3 protein exerts the ability to modulate the apoptotic and anti-tumor properties after being delivered by recombinant adenovirus into macrophages [22]. In our studies, a fusion protein of IRF-3 and C-terminal sequences (310 a.a.) of mouse estrogen receptor was used to establish the stable Huh7.5-IRF3ER cell line. In previous studies, mouse estrogen receptor was effective at inducing dimerization of STAT1 and STAT3 fusion proteins after 4-HT treatment [27,23,29]. In these studies from us and others, 4-HT was titrated to a concentration of 1 μM that achieved the highest expression of STAT1ER and STAT3ER dimerization and the strongest inhibitory effects on HCV RNA replication [23,29]. In our studies, 4-HT-treatment alone was also demonstrated to have no anti-HCV effects [23]. In this study, similar sequences from the mouse ER C-terminal domain were fused to the C-terminus of the IRF-3 gene. In Figure 1, Western blotting with anti-IRF-3 antibody was used to detect the IRF-3 as well as the IRF-3ER monomer and dimer proteins. Lane 1 shows endogenous IRF3 protein (56.1 kd) but no IRF-3ER fusion protein in Huh 7.5 cells treated with 4-HT. Lane 2 shows both IRF-3 and IRF-3ER (monomer) (90 kd) in Huh7.5-IRF3ER cells without 4-HT treatment. Lane 3 shows that 4-HT treatment of Huh7.5-IRF3ER cells induces IRF-3ER fusion protein dimer formation (180 kd) in addition to IRF-3 protein and IRF-3ER monomers. The density of IRF-3ER dimers was less than the density of IRF-3ER monomers (Figure 1, lane 3), which could be explained by the denaturing conditions used in the analysis as suggested in our previous report, including SDS-polyacrylamide gel electrophoresis, RIPA lysis buffer, and boiling during Western blotting [27]. Interestingly, a small amount of IRF-3ER dimer formation was detected in Huh7.5-IRF3ER cells without 4-HT treatment (Figure 1, lane 2). This may be due either to auto-dimerization of IRF-3ER or dimer formation induced by trace estrogen in the tissue culture medium. Multiple forms of the IRF-3ER fusion protein were also detected (Figure 1, lane 3). Our data indicates the IRF-3ER fusion protein approach is an effective means to achieve IRF-3 homodimerization with 4-HT treatment.

### Expression of IFNs after activation of the IRF-3ER fusion protein

**Figure 2:**
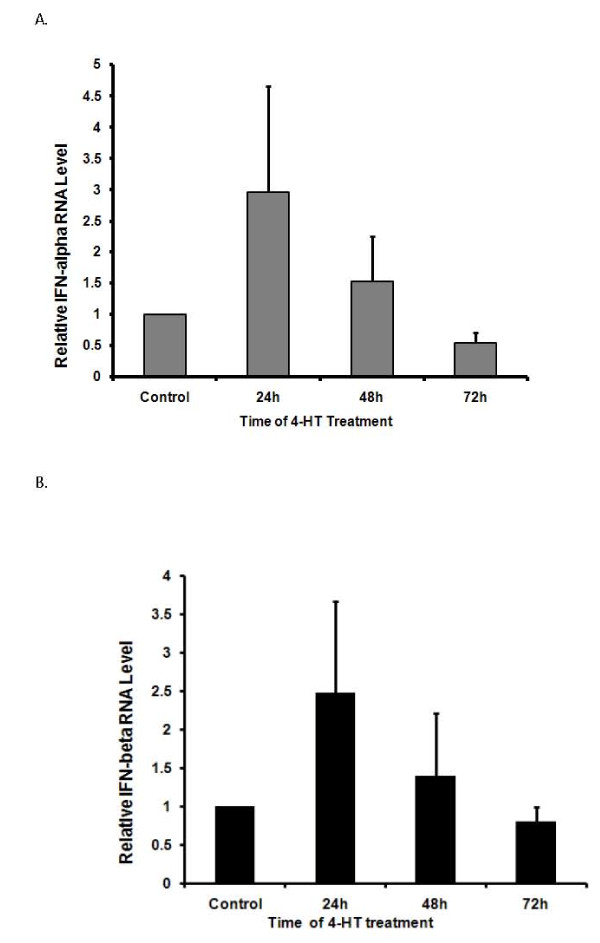
**Expression of IFN-α and IFN-β in Huh7.5-IRF3ER cells with 4-HT treatment**. Huh7.5-IRF3ER cells were treated with 4-HT for 72, 48, and 24 hours prior to collecting cellular lysates. Control is Huh7.5-IRF3ER cells that did not receive 4-HT treatment for 72 hours. Total cellular RNA was isolated for detecting IFN-α or IFN-β RNA by real-time PCR. **A**. Expression of IFN-α in Huh7.5-IRF3ER cells. **B**. Expression of IFN-β in Huh7.5-IRF3ER cells.

**Figure 3:**
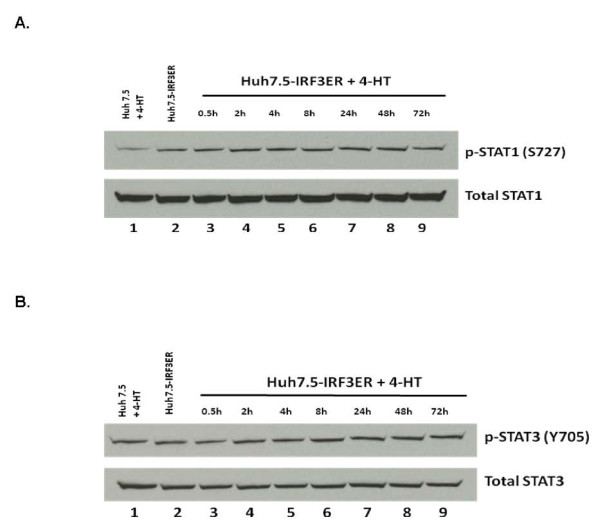
**Phosophorylation of STAT1 and STAT3 proteins in Huh7.5-IRF3ER cells**. Huh 7.5 and Huh7.5-IRF3ER cells were used to detect phosophorylation of STAT1 and STAT3 proteins. Huh7.5-IRF3ER cells were treated without or with 4-HT to induce IRF-3ER dimerization. Total protein was extracted and analyzed by Western blotting with anti-p-STAT1 (S727) antibody, anti-STAT1 antibody (**Figure A**); anti-p-STAT3 (Y705) antibody and anti-STAT3 antibody (**Figure B**), respectively. Lane 1, Huh7.5 cells with 4-HT; lane 2, Huh7.5-IRF3ER without 4-HT; lane 3 to lane 9, Huh7.5-IRF3ER with 4-HT induction from 0.5 to 72 hours.

Due to deficient RIG-I gene function in Huh 7.5 cells, virus infection will not lead to IRF-3 activation and IFN secretion [21]. This phenomenon allows us to study IRF-3 gene function against HCV infection by establishing a stable Huh7.5-IRF3ER cell line. Fusion proteins of STAT1 and STAT3 with the mouse estrogen receptor provided a useful means to study dimerization of those proteins and resulting in anti-HCV status [27,23]. In this study, the IRF-3 gene was fused with same C-terminal sequences of mouse estrogen receptor as reported [27,23] for inducing IRF-3ER dimerization by 4-HT treatment. Expression of type I IFNs (α and β) was examined after 4-HT treatment by real-time PCR. In Figure 2A and 2B, IFN-α and IFN-β increased and peaked 24 hours after 4-HT induction. To further demonstrate the biological activities of IFN-α and IFN-β after IRF-3ER dimerization, Western blotting was used to detect phosphorylated STAT1 and STAT3. In Figure 3A, phosphorylated STAT1 was detected with an antibody against STAT1 (S727) in Huh 7.5 and Huh7.5-IRF3ER cells (Figure 3A, lane 1, 2, 3). Different amounts of phosphorylated STAT1 were observed in both Huh 7.5 cells and Huh7.5-IRF3ER cells (Figure 3A, lane 1 and 2). There were no appreciable time-dependent differences in phosphorylated STAT1 in Huh7.5-IRF3ER cells with or without 4-HT treatment (Figure 3A, lane 2 to 9). This observation is consistent with the auto-dimerization of IRF-3ER fusion protein (Figure 1, lane 2) to produce IFNs (controls of Figure 2A and 2B). In Figure 3B, phosphorylated STAT3 was examined; there was no difference between Huh 7.5, Huh7.5-IRF3ER cells with or without 4-HT treatment (Figure 3B, lane 1 to 9). This phenomenon could be explained by the constant activation of IRF-7 to induce expression of IFN-α which activates the type I IFN pathway through STAT3 phosphorylation [10]. Total STAT1 and STAT3 proteins was used as internal controls and demonstrated no differences with or without 4-HT treatment on Huh7.5-IRF3ER cells or Huh 7.5 cells (Figure 3A and 3B, lane 1 to 9).

### Inhibitory effects of IRF-3ER dimerization on HCV JFH-1 virus replication

**Figure 4:**
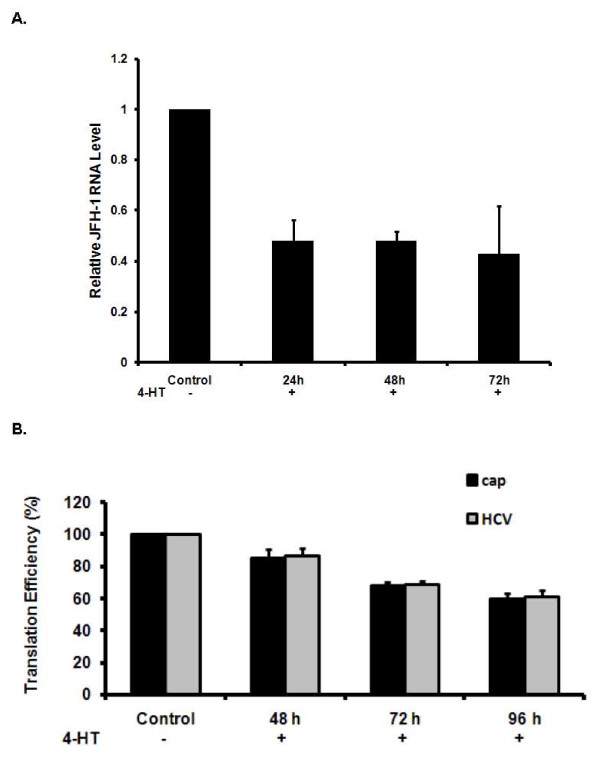
**Anti-HCV effects of the IRF-3ER fusion protein in Huh7.5-IRF3ER cells**. **A**. **Inhibitory effects of IRF-3ER dimerization on HCV RNA replication**. Huh7.5-IRF3ER cells were inoculated with HCV JFH-1 stock at a MOI 0.5 for 14 days and then 4-HT (1 μM) was added at 72, 48, and 24 hours prior to end-point for sample collection. Control indicates the JFH-1 infected Huh7.5-STAT1ER cells without 4-HT treatment for 72 hours. HCV JFH-1 RNA levels were measured by quantitative real-time PCR in triplicate. Relative JFH-1RNA level was calculated as proportion of control (1.0). The data is presented after normalization with an internal GAPDH control. The error bars indicate the variation present in three independent assays. **B. Inhibitory effects of IRF-3ER dimerization on HCV IRES-mediated translation**. Huh7.5-IRF3ER cells were transfected with the plasmid pRL-HL. After 24 hours of incubation at 37°C and 5% CO_2_, transfected Huh7.5-STAT1ER cells were treated with 4-HT for 48, 72, and 96 hours for analyses of luciferase activity. Control Huh7.5-IRF3ER cells received the pRL-HL cDNA plasmid but did not receive 4-HT treatment. Translation efficiency for each sample was calculated as proportion of control (100%). The error bars indicate the variation of three independent assays.

Huh7.5-IRF3ER cells were further examined for its inhibitory effects on HCV JFH-1 viral replication after 4-HT treatment. Huh7.5-IRF3ER cells were inoculated with 0.5 MOI of JFH-1 virus stock and cultured for 14 days to achieve full HCV JFH-1 infected Huh7.5-IRF3ER cell state. The infected Huh7.5-IRF3ER cells were treated with 4-HT (1 μM) at the indicated times and harvested at the last-sample collection point for analysis of HCV RNA by real-time PCR. The infected Huh7.5-IRF3ER cells were used as control without 4-HT treatment for 72 hours. In Figure 4A, HCV JFH-1 replication decreased to 50% of control after 24 hours of 4-HT treatment. This data indicates that IRF-3ER dimerization after 4-HT treatment has inhibitory effects on HCV JFH-1 replication and was correlated with the production of IFN-α and IFN-β (Figure 2A and 2B). To further separate HCV JFH-1 viral RNA replication and viral translation, the plasmid pRL-HL, containing Cap-dependent Renilla luciferase translation and HCV IRES-mediated Firefly luciferase translation start sites, was used in this study [25]. After transfection of pRL-HL, Huh7.5-IRF3ER cell lysates were harvested at various times after 4-HT treatment for analysis of luciferase activity. In Figure 4B, both Cap-dependent and HCV IRES-mediated translation was reduced in Huh7.5-IRF3ER cells after 4-HT treatment in a time-dependent fashion. This data shows strong evidence that activation of the IRF-3ER fusion protein not only inhibits JFH-1 viral RNA replication but also inhibits Cap-dependent and HCV IRES-mediated translation.

### Expression of ISGs in Huh7.5-IRF3ER cells

**Figure 5:**
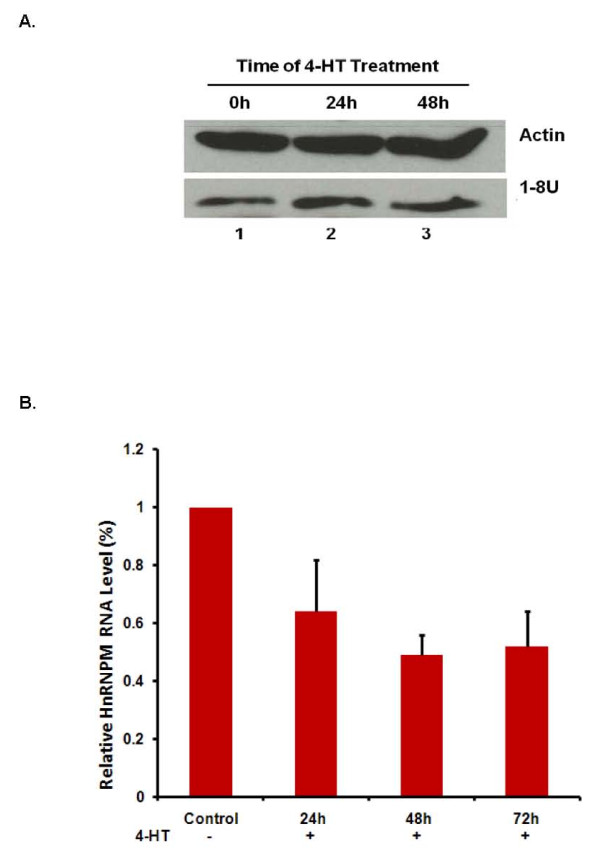
**Expression of 1-8U and hnRNP M genes in Huh7.5-IRF3ER cells**. **A**. **Gene expression of 1-8U in Huh7.5-IRF3ER cells**. Huh7.5-IRF3ER cell lysates were harvested at 0, 24, and 48 hours after adding 4-HT to induce IRF-3ER dimerization. Total protein was extracted and analyzed by Western blotting with an anti-1-8U antibody. Lane 1, Huh7.5-IRF-3ER cell lysate from cells treated with 4-HT at 0 hours; lane 2 and 3, Huh7.5-STAT1ER cell lysates from cells treated with 4-HT for 24 and 48 hours. Actin protein was used as an internal control. **B**. **Expression of hnRNP M in Huh7.5-IRF3ER cells**. Huh7.5-IRF3ER cells were treated by 4-HT (1 μM) for 72, 48, and 24 hours prior to collecting cell lysates. Control is mock treatment of 4-HT for 72 hours. Expression of hnRNPM and GAPDH (used as internal control) mRNA was measured using Taqman primers from Applied Biosystems in triplicate. Relative hnRNP M RNA levels were calculated as a proportion of the control (1.0).

All of the IFN types activate JAK/STAT pathways, regulating the expression of over 300 ISGs in order to achieve their anti-viral effects [30]. In our previous studies, we demonstrated a novel pathway by which IFN inhibits HCV IRES-mediated translation through up-regulating 1-8U gene expression [27] and down-regulating expression of the hnRNP M gene (unpublished data). In this study, we measured 1-8U and hnRNP M expression in Huh7.5-IRF3ER cells with and without 4-HT treatment. In Figure 5A, the 1-8U protein was detected by Western blotting and was up-regulated in Huh7.5-IRF3ER cells after 4-HT treatment. Due to auto-dimerization of IRF-3ER fusion protein in Huh7.5-IRF3ER cells, the fold-induction of 1-8U protein (Figure 5A, lane 1, 2, and 3) is not as robust as described in our previous report in which the STAT1 gene was activated [27]. Real-time quantitative reverse-transcription PCR was used to detect and measure hnRNP M mRNA expression in Huh7.5-IRF3ER cells. After 4-HT treatment, hnRNP M mRNA levels were down-regulated in a time-dependent fashion (Figure 5B). This data confirms that activation of the IRF-3ER fusion protein triggers a cellular anti-HCV state through inducing IFNs production and regulating ISG expression.

## Discussion

Host immunity, including innate immunity and adaptive immunity, is an important and complicated system dedicated to the task of defending the host from microbial infection and cancer development. Innate immunity provides an immediate (first line) reply to a microbial infection, specifically for viral infections, while also controlling the later antigen-specific adaptive response. A key aspect of the antiviral innate immune response is the synthesis and secretion of type I INFs (α and β), which exhibit antiviral, anti-proliferative, and immunomodulatory functions. Two key steps are required to elicit an effective antiviral innate immune response: a. detection of the invading virus by immune system receptors; b. initiation of protein signaling cascades that regulate the synthesis of IFNs. Viruses are highly infectious pathogens that depend on host cellular machinery for survival and replication. Most viral infections, like the common cold caused by Rhinoviruses, are efficiently resolved by the host innate and adaptive immune system. For other viral infections, such as chronic hepatitis B or C viral infection, the host innate and adaptive immunity response is unable to clear them effectively and they become persistent infections. Several families of PPRs have been demonstrated to inspect the cellular micro-environment for microbial infection to target the pathogen-associated molecular patterns (PAMPs), a conserved structural moiety essential for microbial survival. Toll-like receptors (TLRs 3, 4, 7, 8, and 9) in addition to RIG-I are major PPRs that recognize different types of virally-derived nucleic acids or intracellular dsRNA to initiate signaling cascades leading to production of type I IFNs (details in reviews [11,31,32]). The mechanisms by which different viruses induce a unique IFN-mediated antiviral response appear to require selective activation of members of the IRF family of proteins (IRF-1 to IRF-9). Thus far, IRF-3 and IRF-7 have been shown to be major regulators of IFN gene expression [33,10].

The type I IFNs, represented by multiple subtypes of IFN-α in addition to one subtype IFN-β, are key cytokines in this process, mounting an immediate antiviral response as well as adaptive immunity. IFN-mediated anti-viral effects are carried out using different mechanisms that are dependent on the type of viral infection, but these anti-viral effects are all dependent on IRF-3 activation [35,34,33,21,7]. Activation of IRF-3 proteins appears to recruit the Tank Binding Kinase 1 (TBK1) and inhibitor of IκB-related kinase epsilon (IKKε) [7] through their interaction with the RIG-I RNA helicase [36], resulting in phosphorylation of IRF-3, its dimerization, nuclear translocation, and transcriptional activation through binding to IFN-stimulated response elements (ISREs) [37]. Activated IRF-3 interacts with nuclear factor-κB (NF-κB) and transcriptional factor-2/c-Jun to form a transcriptionally active enhanceosome complex on *IFNA1 *and *IFNB *gene promoters.

In our studies, we utilized an IRF-3/mouse ER fusion protein expressing plasmid in order to achieve IRF-3ER activation in a cytokine/receptor-independent fashion. Our results demonstrated that IRF-3ER homodimers (Figure 1, lane 3) triggered the downstream pathways to produce IFN-α and IFN-β (Figure 2A and 2B). The anti-HCV effects, induced by 4-HT in Huh7.5-IRF3ER cells, were achieved by decreasing HCV RNA replication and HCV IRES-mediated translation. This is consistent with our previous studies which achieved activation of STAT1/and STAT3/mouse ER fusion proteins. Activation of the IRF-3ER fusion protein by 4-HT treatment provides strong evidence that this is necessary and sufficient to increase IFN-α and IFN-β expression in Huh7.5-IRF3ER cells (Figure 2A and 2B). Our data showing that IRF-3ER activation triggers the downstream pathway, activating the JAK/STAT pathway and regulating ISG expression. Detection of p-STAT1 (S727) and p-STAT3 (Y705) in Huh7.5-IRF3ER cells provides a strong evidence for activation of Jak/STATs pathway by IFNs (Figure 3A and 3B). Although the mechanism of IFN action against HCV replication has not been well defined, recent studies suggest that IFNs have a great impact on HCV replication by interrupting HCV IRES-mediated translation [38,12]. Clinical data confirmed these findings in a study of HCV IRES-mediated translation in chronic HCV patients receiving IFN treatment, in which the efficiency of HCV IRES-mediated translation was reduced in IFN-treated HCV patients [39,40]. In our study, the inhibitory effects of HCV RNA replication and HCV IRES-mediated translation were confirmed in Huh7.5-IRF3ER cells after treatment with 4-HT (Figure 4A and 4B).

Here, we present data demonstrating that activation of the IRF-3 gene restores IFN production in RIG-I deficient Huh 7.5 cells. The anti-HCV effects were achieved in Huh7.5-IRF3ER cells by decreasing both HCV RNA replication and HCV IRES-mediated translation. Recently, two new genes, 1-8U and hnRNP M, were isolated in our studies due to their ability to modulate cellular Cap-dependent and HCV IRES-mediated translation and regulated by STAT1 pathway activation.

## Competing interests

The authors declare that they have no competing interests.

## Authors' contributions

YL was a post-doctor trainee who carried out most experiments in this paper at the University of Florida-Jacksonville. DH carried out part of real-time PCR assay. XL is PI and others are his cooperators in Virology, Immunology, and Hepatology. All authors read and approved the final manuscript.
